# Intra-urban variation in tuberculosis and community socioeconomic deprivation in Lisbon metropolitan area: a Bayesian approach

**DOI:** 10.1186/s40249-022-00949-1

**Published:** 2022-03-24

**Authors:** Olena Oliveira, Ana Isabel Ribeiro, Raquel Duarte, Margarida Correia-Neves, Teresa Rito

**Affiliations:** 1 Life and Health Sciences Research Institute (ICVS), School of Medicine, University of Minho, 4710-057 Braga, Portugal; 2 ICVS/3B, PT Government Associate Laboratory, University of Minho, 4710-057 Braga, Portugal; 3 Epidemiology Research Unit (EpiUnit), Institute of Public Health, University of Porto, Porto, Portugal; 4 Public Health and Forensic Sciences, and Medical Education Department, Faculty of Medicine, University of Porto, Porto, Portugal; 5 Laboratory for Integrative and Translational Research in Population Health (ITR), Rua das Taipas 135, 4050-600 Porto, Portugal; 6 Institute of Biomedical Sciences Abel Salazar, University of Porto, Porto, Portugal; 7 Division of Infectious Diseases, Department of Medicine Solna, Karolinska Institutet, Stockholm, Sweden; 8 Centre of Molecular and Environmental Biology (CBMA), Department of Biology, University of Minho, 4710-057 Braga, Portugal; 9 Institute of Science and Innovation for Bio-Sustainability (IB-S), University of Minho, 4710-057 Braga, Portugal

**Keywords:** Tuberculosis, Multidrug-resistant tuberculosis, Bayesian, Spatial analysis, Socioeconomic deprivation

## Abstract

**Background:**

Multidrug resistant tuberculosis (MDR-TB) is a recognized threat to global efforts to TB control and remains a priority of the National Tuberculosis Programs. Additionally, social determinants and socioeconomic deprivation have since long been associated with worse health and perceived as important risk factors for TB. This study aimed to analyze the spatial distribution of non-MDR-TB and MDR-TB across parishes of the Lisbon metropolitan area of Portugal and to estimate the association between non-MDR-TB and MDR-TB and socioeconomic deprivation.

**Methods:**

In this study, we used hierarchical Bayesian spatial models to analyze the spatial distribution of notification of non-MDR-TB and MDR-TB cases for the period from 2000 to 2016 across 127 parishes of the seven municipalities of the Lisbon metropolitan area (Almada, Amadora, Lisboa, Loures, Odivelas, Oeiras, Sintra), using the Portuguese TB Surveillance System (SVIG-TB). In order to characterise the populations, we used the European Deprivation Index for Portugal (EDI-PT) as an indicator of poverty and estimated the association between non-MDR-TB and MDR-TB and socioeconomic deprivation.

**Results:**

The notification rates per 10,000 population of non-MDR TB ranged from 18.95 to 217.49 notifications and that of MDR TB ranged from 0.83 to 3.70. We identified 54 high-risk areas for non-MDR-TB and 13 high-risk areas for MDR-TB. Parishes in the third [relative risk (RR) = 1.281, 95% credible interval (CrI): 1.021–1.606], fourth (RR = 1.786, 95% CrI: 1.420–2.241) and fifth (RR = 1.935, 95% CrI: 1.536–2.438) quintile of socioeconomic deprivation presented higher non-MDR-TB notifications rates. Parishes in the fourth (RR = 2.246, 95% CrI: 1.374–3.684) and fifth (RR = 1.828, 95% CrI: 1.049–3.155) quintile of socioeconomic deprivation also presented higher MDR-TB notifications rates.

**Conclusions:**

We demonstrated significant heterogeneity in the spatial distribution of both non-MDR-TB and MDR-TB at the parish level and we found that socioeconomically disadvantaged parishes are disproportionally affected by both non-MDR-TB and MDR-TB. Our findings suggest that the emergence of MDR-TB and transmission are specific from each location and often different from the non-MDR-TB settings. We identified priority areas for intervention for a more efficient plan of control and prevention of non-MDR-TB and MDR-TB.

**Graphical Abstract:**

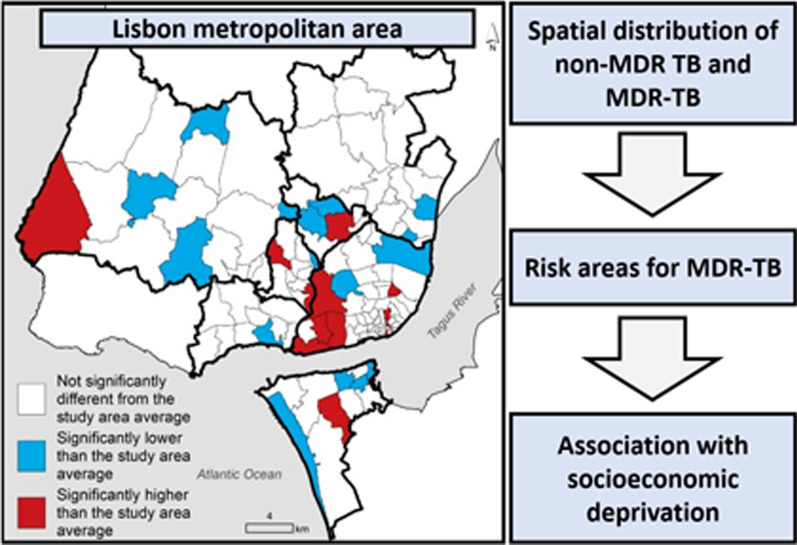

**Supplementary Information:**

The online version contains supplementary material available at 10.1186/s40249-022-00949-1.

## Background

The global strategy for tuberculosis (TB) control aims at ending the TB epidemic. The targets established by the World Health Organization (WHO) are to reduce, until 2035, by 95% the number of deaths from TB and 90% the incidence rate of the disease compared with 2015 [1, 2].

However, despite great advances and achievements in the control of TB, this disease is one of the top 10 causes of death worldwide and was the leading cause of death from a single infectious agent [3] until the coronavirus disease 2019 pandemic [4].

The distribution of TB across the world is not homogeneous. In 2019, most TB cases were in the regions of South-East Asia (44%) and Africa (25%). While the Americas and Europe accounted 2.9% and 2.5% of the cases [3]. Globally, there were 130 cases per 100,000 population. Lesotho and South Africa stood out as the countries with a higher incidence rate: 654 and 615 cases per 100,000 population respectively [3]. Countries with low incidence of TB (< 10 cases per 100,000 population) are considered well placed to target TB elimination. While these countries are mostly located in the Americas and Europe, Portugal is not in that situation [3].

In Portugal, efforts have been made aiming at that goal, and, consequently the TB notification rate in 2019 was 18.0 per 100,000 population, continuing a downward trend with an annual decline in TB notification rate of 3.9% in the last 5 years [5]. However, in the same period (2015–2019), the average annual decrease in the notification rate within the whole European Union was 5.1%, leading to an average notification rate of 9.6 per 100,000 in 2019 [6], nearly half of the observed in Portugal [5]. This highlights that in Portugal it is still essential to implement new measures to accelerate the decrease of TB burden to be in line with the remaining European countries.

Two of the challenges that arose against the goal of TB eradication are the emergence and transmission of multi-drug resistance strains in the last few years and the existence of risk groups within the population, both contributing to a persistence of transmission chains and a decreased success of the treatment leading to poorer outcomes. A well-known major factor that contributes to the increased burden of TB are social determinants. Social determinants, which include conditions in which people are born, grow, live, work and age, are mostly responsible for health inequities [7]. Conditions such as low income, poor housing, poor education and unemployment, defined as socioeconomic deprivation [8, 9], are associated with worse health [10], and are also generally perceived as increased risk factors for TB [9, 11]. For effective TB control and prevention, actions to address underlying social determinants beyond efforts in the health sector are also required [1, 2].

Several studies about TB inequalities revealed higher rates of TB associated with socioeconomic deprivation, namely low income, high crowding, less education and high unemployment in the United States [12], population density and socioeconomic conditions in Brazil [13] and socioeconomic deprivation in England [14]. While socioeconomic deprivation is a known fact that correlate with burden of TB in large geographic areas, few studies addressed the heterogeneity of those conditions [15] across a major metropolitan area, where the existence of pockets of deprived groups of individuals with a higher burden of TB might be a challenge for a detailed monitoring of the disease and lead to misplaced public health efforts. Furthermore, there is an absence of studies detailing the role of MDR-TB transmission in these complex urban set-ups under heterogeneous socioeconomic deprivation. To measure socioeconomic deprivation, a cross-national ecological deprivation index was created in 2016 [16] for the small areas of England, France, Italy, Portugal, and Spain—the European Deprivation Index (EDI). The EDI has been used to investigate inequalities in several health outcomes in Portugal [17–19] and other countries [20, 21], including TB inequalities [22]. In this last mentioned investigation [22], the authors evaluated the association between TB notification and socioeconomic deprivation across municipalities in Portugal. In that study, the TB notification rate was not significantly associated with the overall composite EDI. Still, it was significantly associated with some of its component variables, such as the proportion of manual workers and the percentage of unemployed. In contrast, the variable “proportion of residents with low education level” showed an inverse relationship.

In our previous study [23], we analyzed the spatial distribution of the MDR-(resistant to at least isoniazid and rifampicin) and non-MDR-TB (all other TB) cases across municipalities in Portugal, and we demonstrated that this distribution was very heterogeneous, highlighting the relevance of applying such methodology to the Portuguese territory. We identified 36 high-risk areas for non-MDR-TB, 8 of which were also high-risk areas for MDR-TB in a total of 278 defined areas. Seven of these 8 high-risk areas for both MDR-TB and non-MDR-TB are located in the Lisbon metropolitan area. Previous genetic studies revealed the existence of two large clusters that are being continuously transmitted within the community in the last three decades [24–26]. Such uneven geographical distribution shows that we need to include geographical criteria in the screening and prevention programs for better and more efficient control. However, municipalities in Portugal still hold considerable heterogeneity between them, ranging from as few as 1,830 inhabitants to over 500,000 inhabitants.

Consequently, we might have failed to detect important spatial inequalities when considering municipalities as the unit for the analysis, and great heterogeneity could be present within these broad urban areas. Under the Precision Public Health paradigm, adapting targets and interventions to the local context is pivotal to effective disease control [27]. Therefore, it is important to monitor disease variation using finer geographical scales [28] using appropriate statistical methods capable of dealing with the Small Numbers Problem and spatial autocorrelation.

The knowledge on the distribution of the disease in specific geographical areas or subpopulations with especially high TB burden is crucial for local adjustment of control measures and to the redistribution of resources necessary for better control and TB prevention [2]. This knowledge can be achieved through disease mapping and spatial analysis that are highly effective approaches to investigate the detailed geographical variations in TB incidence [29] and to identify high- and low-risk areas [22, 23, 30].

While there are several studies establishing the association between TB and socioeconomic deprivation, less is known about its influence on MDR-TB in particular, and we do not know how the importance of socioeconomic deprivation in TB incidence compares between MDR-TB and in the more known non-MDR-TB. In addition, as far as we are aware, no study addressed the association between area-level deprivation and MDR-TB. Thus, to address these gaps, we analyzed the spatial distribution of notification of both non-MDR-TB and MDR-TB across parishes of the seven major municipalities of the Lisbon metropolitan area, which have been previously identified as high-risk areas for non-MDR-TB and MDR-TB (*Almada, Amadora, Lisboa, Loures, Odivelas, Oeiras, Sintra*). We also assessed the correlation between the spatial distributions of non-MDR-TB and MDR-TB notification and estimated the association between non-MDR-TB and MDR-TB and socioeconomic deprivation.

## Methods

### Study area and data collection

We conducted an ecological retrospective study at parish level [31]. Our study focus on the Lisbon metropolitan area, where a previous study identified seven of the eight high-risk areas for TB and MDR-TB in Portugal [23]. Within the 18 municipalities of the Lisbon metropolitan area, the 7 with the greater number of resident population *are Almada, Amadora, Lisboa, Loures, Odivelas, Oeiras, Sintra*, ranging between 147,563 (*Odivelas*) and 542,440 (*Lisboa*) inhabitants. In these seven municipalities reside 63.4% of the Lisbon metropolitan area inhabitants. These seven municipalities are subdivided into 127 parishes, where the number of resident population ranges between 355 and 79,805 inhabitants.

We analyzed all TB cases, which included new and relapse cases of TB, notified from January 2000 until December 2016 from the seven municipalities using the Portuguese TB Surveillance System (SVIG-TB). We selected all TB cases, and we further divided these into two groups: MDR-TB and non-MDR-TB cases. We obtained notifications of MDR-TB and non-MDR-TB by each of the 127 parishes. Population counts by parishes were obtained from Statistics Portugal (https://www.ine.pt/) for the study period.

### Socioeconomic deprivation

The European Deprivation Index for Portugal (EDI-PT) was used as an indicator of socioeconomic deprivation in the 127 analyzed parishes. The EDI-PT was composed by eight census variables, and their score resulted from the equation presented in Table 1 [19].

**Table 1:** The equation for the calculation of the EDI-PT

	Variables
EDI-PT score=	% non-owned households × 1.191 + % households without indoor flushing × 1.729 + % household with 5 rooms or less × 0.964 + % blue-collars × 0.370 + % residents with low education level × 0.511 + % non-employers × 0.620 + % unemployed looking for a job × 0.268 + % foreign residents × 1.038

The EDI was standardized and classified into five quintiles (from Q1, the least deprived, to Q5, the most deprived).

### Statistical analysis

We used hierarchical Bayesian spatial models to estimate the relative risk and notification rates in each area and delimitate high risk and low-risk areas. To minimize the effect of random fluctuations associated with a small number of cases, and because we found no substantial differences in the geographical distribution of non-MDR-TB and MDR-TB across our study period, we considered the average rates of the 17-year study period. We assumed that the response variable, cases of TB $({O}_{i})$ in each $i$th area, follows a Poisson distribution where ${E}_{i}$. Is the expected number of cases and ${\theta }_{i}$ the relative risk (RR) (Eq. 1). We used the TB notification rates of the whole study area (dividing the sum of the notifications and populations of *Almada, Amadora, Lisboa, Loures, Odivelas, Oeiras, Sintra*) as a reference to compute the expected number of cases. The expected number of cases was obtained by summing the product of the notification rates of the reference population (those of the study area) by the population of each parish (*n* = 127) of the study area.

1 $${O}_{i}\sim Poisson \left({E}_{i}, {\theta }_{i}\right)$$

2.1 $$log\left({\theta }_{i}\right)=\alpha + {s}_{i}$$

Here $\alpha$ is an intercept quantifying the average number of TB cases in the 127 areas (parishes). The area specific effect ${s}_{i}$ was modelled considering a BYM model [32] with a parameterization suggested by Dean and colleagues [33] (Eq. 2.2).

2.2 $${s}_{i}=\tau (\sqrt{\varphi }*{u}_{i}+\sqrt{1-\varphi }*{v}_{i})$$

where ${u}_{i}$ is the structured effect and ${v}_{i}$ is the unstructured effect. The ${u}_{i}$ effect was scaled to render the model more intuitive and interpretable [34], so that $\varphi$ expresses the proportion of the spatial effect due to the structured part and 1/$\tau$ is the marginal variance of ${s}_{i}$.

Additionally, we used the function ‘excursions’ to delimitate high risk and low risk areas [22, 35, 36]. High-risk areas are those whose RR is significantly above 1 (i.e., above the study area average) and low risk areas are those whose RR is significantly below 1 (i.e., below the study area average). This method uses the posterior joint distribution computed from the Integrated Nested Laplace Approximation (INLA). It considers the dependence structure, allowing the accurate identification of areas where the RR is greater than 1.

To analyse the correlation between MDR-TB and non-MDR-TB, the Pearson correlation coefficient [r and corresponding 95% credible intervals (95% CrI)] was computed. To facilitate interpretation, RR was converted into rates per 10,000 inhabitants.

We also used the above-mentioned models to evaluate the association between non-MDR- and MDR-TB and the EDI-PT. Association was expressed in RR that represents the ratio between the risk of non-MDR- and MDR-TB of a deprivation quintile and the risk of the reference class (Q1, the least deprived). An RR would be considered significantly higher or lower if 95% CrIs did not include the value 1. RRs and 95% CrIs were derived from their posterior means and quintiles. Posterior distributions were obtained using the INLA, which was implemented in the R INLA library [37].

The RR and high and low risk areas were mapped using ArcMap release 10.7.1. (Environmental Systems Research Institute, Redlands, CA, USA). For mapping, we used the official reference system for Continental Portugal—PT-TM06/ETRS89 (epsg: 3763)—whose datum is the European Terrestrial Reference System 1989 (ellipsoid: GRS 1980) [38, 39].

#### Ethical considerations

Ethical approval and informed consent were not required, as the patient data, collected for an official national surveillance system, were anonymized in accordance with the ethical research guidelines in Portugal. Authorization for its use in the present manuscript was given by the National Program for Tuberculosis.

## Results

### Heterogeneous distribution of non-MDR and MDR TB in Lisbon

From 2000 to 2016, 13,903 cases of non-MDR-TB and 282 cases of MDR-TB were notified in the 127 parishes of the seven investigated municipalities of Lisbon metropolitan area (*Almada, Amadora, Lisboa, Loures, Odivelas, Oeiras, Sintra*). Pulmonary TB represented 71% of non-MDR-TB cases and 90% of MDR-TB cases were pulmonary, the median age of the patients was 39 years in both groups, and 65% of the patients with non-MDR-TB and 68% of the patients with MDR-TB were male.

The overall crude non-MDR-TB notification rate was 77.40 notifications per 10,000 population (95% CrI: 76.12–78.68) and the overall crude MDR-TB notification rate was 1.58 notifications per 10,000 population (95% CrI: 1.40–1.76).

**Fig. 1:**
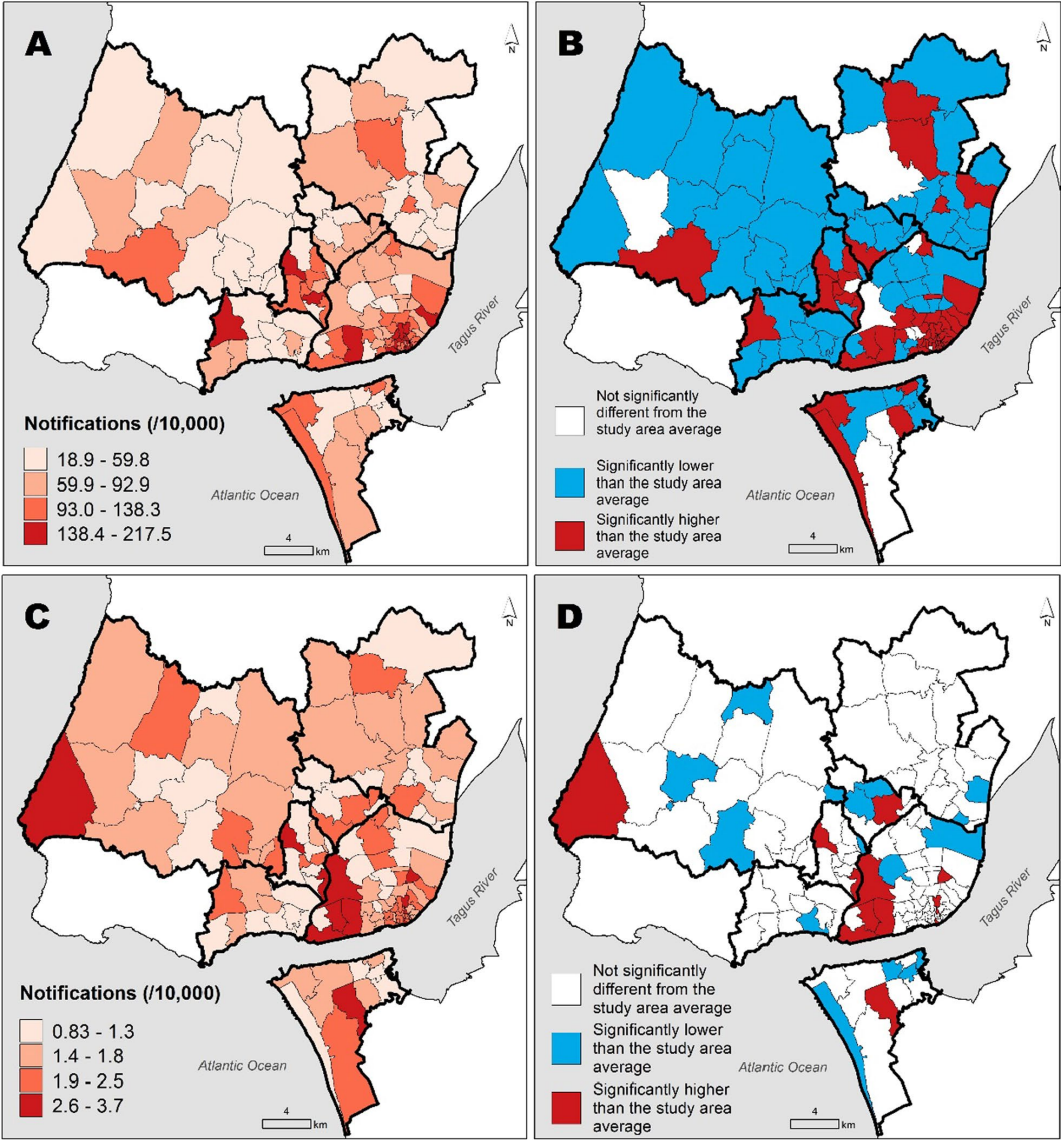
Spatial distribution of the notification rates of non-MDR-TB (**A**) and the corresponding delimitation of the high- and low-risk areas (**B**). Spatial distribution of the notification rates of MDR-TB (**C**) and the corresponding delimitation of the high- and low-risk areas (**D**) across the 127 parishes of the *Almada, Amadora, Lisboa, Loures, Odivelas, Oeiras and Sintra* municipalities, 2000–2016. High-risk areas are those whose relative risk is significantly above 1 (i.e., above the study area average); low risk areas are those whose relative risk is significantly below 1 (i.e., below the study area average)

The spatial distribution of the notification rates of non-MDR-TB and MDR-TB is depicted in Fig. 1A and C with the delimitation of the high- and low-risk areas given in Fig. 1B and D. The notification rates per 10,000 population of non-MDR TB ranged from 18.95 to 217.49 notifications and that of MDR TB ranged from 0.83 to 3.70.

We identified 54 high-risk areas for non-MDR-TB and 13 high-risk areas for MDR-TB. Among all these areas, only 8 were simultaneously high-risk areas for non-MDR- and MDR-TB that corresponds to 15% (8/54) of the non-MDR-TB and 62% (8/13) of MDR-TB high-risk areas. That is to say, 38% (5/13) of MDR-TB high-risk areas were not risk areas for non-MDR-TB (Additional file 1: Table S1).

The correlation between MDR-TB and non-MDR-TB notification ratios was moderate (ρ = 0.555; 95% CrI: 0.422–0.665).

### Socioeconomic deprivation associated with MDR and non-MDR TB risk

Furthermore, we found that the EDI-PT was positively and significantly associated with both non-MDR-TB and MDR-TB notifications. Parishes in the third, fourth and fifth quintile of socioeconomic deprivation, the most deprived areas, presented higher non-MDR-TB notifications rates (RR = 1.281, 95% CrI: 1.021–1.606; RR = 1.786, 95% CrI: 1.420–2.241 and RR = 1.935, 95% CrI: 1.536–2.438, respectively) (Table 2). Moreover, it is important to refer that the excess risk of non-MDR-TB increased from 28.1 to 93.5% with increased deprivation from the third to the fifth quintiles of the EDI-PT. Note that 91% of the high-risk areas for non-MDR-TB are parishes in the fifth quintile (most socioeconomic deprived) (Additional file 1: Table S1). Parishes in the fourth and fifth quintile of socioeconomic deprivation also presented higher MDR-TB notifications rates (RR = 2.246, 95% CrI: 1.374–3.684 and RR = 1.828, 95% CrI: 1.049–3.155, respectively) (Table 2). The excess risk of MDR-TB was higher in the fourth quintile (124.6%). Among the high-risk areas for MDR-TB, 23% are parishes in the fourth quintile and 77% in the fifth quintile (Additional file 1: Table S1).

**Table 2:** Associations between non-multidrug-resistant and multidrug-resistant tuberculosis notifications and the European Deprivation Index for Portugal in the 127 parishes of the *Almada, Amadora, Lisboa, Loures, Odivelas, Oeiras and Sintra* municipalities, 2000–2016

EDI-PT	RR (95% CrI)	
	Non-MDR-TB	MDR-TB
Q1—least deprived	1.000 (reference)	1.000 (reference)
Q2	0.999 (0.798–1.250)	1.525 (0.902–2.552)
Q3	1.281 (1.021–1.606)	1.483 (0.893–2.472)
Q4	1.786 (1.420–2.241)	2.246 (1.374–3.684)
Q5—most deprived	1.935 (1.536–2.438)	1.828 (1.049–3.155)

EDI: European Deprivation Index for Portugal; quintiles of EDI; RR: relative risk; CrI: credible interval; MDR-TB: multidrug-resistant tuberculosis

## Discussion

In this study, we analyzed the spatial distribution of notification of non-MDR-TB and MDR-TB over 17 years in 127 parishes of the seven municipalities of the Lisbon metropolitan area with a higher population size (*Almada, Amadora, Lisboa, Loures, Odivelas, Oeiras, Sintra*). The results show significant heterogeneity in the spatial distribution at the parish level, and we found a moderate correlation between MDR-TB and non-MDR-TB notification ratios. In addition, we identified 54 high-risk areas for non-MDR-TB and 13 high-risk areas for MDR-TB. We also showed that notification ratios are positively associated with socioeconomic deprivation. The spatial distribution of the non-MDR-TB and MDR-TB notification rates across parishes in the study area was not homogeneous. A high degree of heterogeneity (up to 11 times difference) was observed in the spatial distribution of the non-MDR-TB notification rates, ranging from 18.95 to 217.49 notifications per 10,000 population. However, the notification rates of MDR-TB showed less variation (up to four times difference), ranging from 0.83 to 3.70 notifications per 10,000 population.

In our previous study, the spatial distribution of non-MDR-TB and MDR-TB notification rates at the municipality level, using a wider geographic scale, was also heterogeneous [23]. Heterogeneity in the geographic distribution of TB has also been reported in previous studies on Portugal [22, 40, 41]. Such trend for a heterogeneous distribution is also observed on several other countries, independently of the geographic scale, reported in a systematic review comparing low- and high-incidence settings [29] and other studies in Bangladesh [42], Moldova [43], China [44] and Ethiopia [45].

In the present study, the correlation between non-MDR-TB and MDR-TB at the parish level was moderate, which corroborates the moderate correlation observed at the municipality level [23] in Portugal. We identified 54 high-risk areas for non-MDR-TB and 13 high-risk areas for MDR-TB, but only 8 were simultaneously high-risk areas for both non-MDR-TB and MDR-TB, which corresponds to 15% of the non-MDR-TB and 62% of MDR-TB high-risk areas. This means that 38% (5/13) of MDR-TB high-risk areas are not risk areas for non-MDR-TB. The high-risk areas are described in Additional file 1: Table S1 and which are referenced as having high population density. This finding suggests that MDR-TB in the study area is mainly due to the transmission of MDR strains in specific parishes regardless of the non-MDR-TB burden. Transmission of MDR strains in the Lisbon metropolitan area has already been analyzed in previous genetic studies [24–26] and being Portugal-born was the major factor associated with MDR-TB recent transmission [26].

Finally, we showed that non-MDR-TB and MDR-TB notification ratios are positively associated with socioeconomic deprivation, measured by EDI-PT. The excess risk of non-MDR-TB gradually increased from 28.1% to 93.5%, with increased deprivation from third to fifth quintiles of the EDI-PT. Notably, 91% of the high-risk areas for non-MDR-TB are parishes in the fifth quintile (most socioeconomic deprived) (Additional file 1: Table S1). This finding is not consistent with the results from a previous assessment at the municipality level across Portugal [22], where an association between TB notification rate and composite EDI was not found. However, in that previous study, TB notification rate was associated with some EDI component variables such as the proportion of manual workers and the percentage of unemployed. In contrast, the variable “proportion of residents with low education level” showed an inverse relationship. This difference between the results of the studies can be explained firstly by using different EDI versions and geographical units with different sizes, being our study an updated improved version. We used the updated EDI-PT version that included variables related to nationality (proportion of foreign residents) and employment condition (proportion of non-employees) [19], whereas in the version used in the previous study, these were absent, but were included variables related with demography (age and sex) and the presence of a shower/bath in the household [22, 46]. Secondly, we assessed the association separately between non-MDR-TB and MDR-TB notification and socioeconomic deprivation across municipalities with high-risk for non-MDR-TB and MDR-TB. In contrast, the authors of the other study assessed the association between all TB notifications and socioeconomic deprivation across all municipalities in Portugal [22].

On the other hand, our findings are consistent with the results from an assessment conducted in England [14] where TB rates were positively associated with small-area levels of socioeconomic deprivation. Moreover, this relationship between area-deprivation and TB rates was much stronger in the UK-born population compared to foreign-born: TB rates in UK-born persons living in the most deprived quintile areas were 2.4 times higher than the least deprived quintile areas, compared to just about 80% higher rates in foreign-born persons for the same comparison.

Regarding the relationship between MDR-TB notification ratios and socioeconomic deprivation, we did not find any study with a similar methodology to compare our results. Our study observed higher MDR-TB notification ratios in parishes in the fourth and fifth quintile of socioeconomic deprivation. However, the higher excess risk of MDR-TB was observed in parishes in the fourth but not on the fifth quintile that was classified as most socioeconomic deprived (124.6% and 82.8%, respectively), contrary to the pattern of risk for non-MDR-TB.

Our findings suggest that socioeconomic deprivation is an important risk factor for both non-MDR-TB and MDR-TB. Nonetheless, other individual factors such as health-related behaviour (e.g. drug or alcohol abuse) and HIV infection must also play an important role in MDR-TB notification rates [23] even in less deprived parishes.

The use of robust statistical methods to characterize geographic patterns across parishes, which allowed the identification of risk in more geographically-defined small areas for non-MDR-TB and MDR-TB, was one of the strengths of this study. This should help in the adjustment of local TB control strategies. Another strength was the use of the good quality surveillance data (as previously demonstrated [47, 48]) that is exported electronically to the ECDC–WHO Regional Office for Europe Joint TB Information System and to The European Surveillance System (TESSy) platform hosted by ECDC [6]. The use of the updated index of socioeconomic deprivation for Portuguese small-areas that allowed the study of the association between non-MDR-TB and MDR-TB notification and socioeconomic deprivation was also another strength. However, we did not assess the prevalence of drug or alcohol abuse, which are in turn related to socioeconomic inequalities [49, 50], and HIV infection among the resident population at the parish level since this information is not available at this geographical level, which can be seen as a study limitation.

## Conclusions

In conclusion, we found heterogeneity and a moderate correlation in the spatial distribution of non-MDR-TB and MDR-TB across parishes in the study area. Our findings suggest that the MDR-TB emergence and transmission has its setting, different from the non-MDR-TB environment for the most part, allowing the identification of specific transmission routes within more precise areas prompting a more effective action of public health agents with support of community institutions. The additional genetic studies of all TB isolates could contribute to a better understanding the dynamics of MDR-TB emergence and transmission in these specific areas. In addition, we found that socioeconomically disadvantaged parishes are disproportionally affected by non-MDR-TB and MDR-TB, again contributing to the identification of priority areas for intervention against non-MDR-TB and MDR-TB adjusted to the socioeconomic needs of the population and a more efficient plan of action. These interventions demand a multidisciplinary and multisectoral approach to reduce vulnerability to the disease in specific social groups.

## Supplementary Information

**Additional file 1: Table S1. **Characteristics of the high-risk areas (RR is significantly above 1, i.e., above the study area average) for non-MDR-TB and MDR-TB, in the *Almada, Amadora, Lisboa, Loures, Odivelas, Oeiras and Sintra *municipalities, 2000–2016.

## Data Availability

The epidemiological and geographical datasets generated during the current study are available from the corresponding author on reasonable request.

## Abbreviations

BYM: Spatial Besag–York–Mollié model
CrI: Credible interval
ECDC: European Centre for Disease Prevention and Control
EDI: European Deprivation Index
EDI-PT: European Deprivation Index for Portugal
INLA: Integrated Nested Laplace Approximation
MDR-TB: Multidrug resistant tuberculosis
RR: Relative risk
SVIG-TB: Portuguese TB Surveillance System
TB: Tuberculosis
TESSy: The European Surveillance System
WHO: World Health Organization
