# Supplementary material for: Intra-urban variation in tuberculosis and community socioeconomic deprivation in Lisbon metropolitan area: a Bayesian approach
Source: Infect Dis Poverty. 2022 Mar 24;11:24. doi: 10.1186/s40249-022-00949-1 (PMC8942608; doi:10.1186/s40249-022-00949-1)
Supplement: Supplementary file 1 — Additional file 1: Table S1. Characteristics of the high-risk areas (RR is significantly above 1, i.e., above the study area average) for non-MDR-TB and MDR-TB, in the Almada, Amadora, Lisboa, Loures, Odivelas, Oeiras and Sintra municipalities, 2000–2016. [file 40249_2022_949_MOESM1_ESM.docx]

**Table S1. Characteristics of the high-risk areas (RR is significantly above 1, i.e., above the study area average) for non-MDR-TB and MDR-TB, in the *Almada, Amadora, Lisboa, Loures, Odivelas, Oeiras and Sintra* municipalities, 2000–2016**

| **PARISHES** | **Population density,**  inhabitants per km² | **Non-MDR-TB (RR)** | **MDR-TB (RR)** | **Residents with low education level** **(%)** | **Foreign residents (%)** | **Non-employers (%)** | **Unemployed looking for a job (%)** | **EDI_**  **SCORE** | **EDI_**  **QUINTILE** |
| --- | --- | --- | --- | --- | --- | --- | --- | --- | --- |
| Ajuda | 5456,9 | 1,786 | 1,779 | 0,47646 | 0,03844 | 0,90203 | 0,15577 | 4,67260 | 5 |
| Alcântara | 3142 | 2,110 | 2,375 | 0,37004 | 0,06634 | 0,89306 | 0,11393 | 4,89874 | 5 |
| Alto do Pina | 12319,6 | nd | 1,862 | 0,28613 | 0,06803 | 0,85478 | 0,10704 | 1,64544 | 4 |
| Alvalade | 14898,5 | 1,199 | nd | 0,03732 | 0,84485 | 0,08675 | 3,10790 | -0,47678 | 3 |
| Anjos | 19180,8 | 2,779 | 1,756 | 0,33767 | 0,14774 | 0,87703 | 0,13604 | 6,33042 | 5 |
| Beato | 7670,9 | 2,810 | nd | 0,45788 | 0,06871 | 0,90461 | 0,16409 | 6,03819 | 5 |
| Benfica | 4639,5 | nd | 1,981 | 0,34420 | 0,04076 | 0,89249 | 0,13302 | 1,99020 | 5 |
| Campolide | 5571,9 | 1,195 | nd | 0,39649 | 0,09153 | 0,87586 | 0,12558 | 5,97800 | 5 |
| Castelo | 6440,7 | 2,259 | nd | 0,51935 | 0,03662 | 0,89844 | 0,20000 | 8,34034 | 5 |
| Charneca | 5866,6 | 1,414 | nd | 0,51918 | 0,08274 | 0,92022 | 0,18068 | 8,06459 | 5 |
| Coração de Jesus | 6607,4 | 1,669 | nd | 0,27178 | 0,10735 | 0,86043 | 0,09652 | 3,10047 | 5 |
| Encarnação | 11920,7 | 2,377 | nd | 0,37372 | 0,08615 | 0,84783 | 0,11072 | 4,94197 | 5 |
| Graça | 16507,9 | 1,389 | nd | 0,42191 | 0,09055 | 0,88490 | 0,14204 | 6,31579 | 5 |
| Madalena | 3400,5 | 2,116 | 2,322 | 0,23313 | 0,12214 | 0,85106 | 0,13761 | 4,31547 | 5 |
| Marvila | 5977,6 | 1,383 | nd | 0,54729 | 0,03257 | 0,92612 | 0,16773 | 5,47007 | 5 |
| Mercês | 16203,5 | 1,727 | nd | 0,34753 | 0,08262 | 0,85926 | 0,13914 | 5,49204 | 5 |
| Pena | 8971,3 | 2,157 | nd | 0,38433 | 0,15002 | 0,88780 | 0,14443 | 8,80063 | 5 |
| Penha de França | 18708,7 | 1,354 | nd | 0,35721 | 0,09859 | 0,88219 | 0,11265 | 5,27659 | 5 |
| Sacramento | 8714,7 | 1,671 | nd | 0,31944 | 0,09569 | 0,82486 | 0,11278 | 4,59893 | 5 |
| Santa Catarina | 17407,1 | 1,664 | nd | 0,33794 | 0,10118 | 0,83314 | 0,12110 | 5,40363 | 5 |
| Santa Engrácia | 9543,1 | 1,152 | nd | 0,40009 | 0,08554 | 0,88978 | 0,12553 | 6,10176 | 5 |
| Santa Justa | 3614 | 2,721 | nd | 0,39381 | 0,33333 | 0,79849 | 0,14069 | 17,15695 | 5 |
| Santa Maria de Belém | 2488,9 | 1,387 | 1,770 | 0,29814 | 0,04262 | 0,83689 | 0,09549 | 0,22581 | 4 |
| Santiago | 9542,4 | 1,401 | nd | 0,42599 | 0,06462 | 0,84921 | 0,10954 | 5,90225 | 5 |
| Santo Condestável | 14847,1 | 1,605 | nd | 0,34523 | 0,05067 | 0,87647 | 0,11472 | 3,88882 | 5 |
| Santo Estevão | 7428,9 | 1,385 | nd | 0,46703 | 0,06486 | 0,90080 | 0,14850 | 7,78507 | 5 |
| Santos-o-Velho | 7569,2 | 1,274 | nd | 0,34084 | 0,08333 | 0,86682 | 0,13052 | 5,05487 | 5 |
| São Cristóvão e São Lourenço | 17190,8 | 1,631 | nd | 0,44657 | 0,15735 | 0,84398 | 0,17005 | 10,35031 | 5 |
| São João | 10087,2 | 1,201 | nd | 0,40436 | 0,07190 | 0,90233 | 0,14702 | 5,53393 | 5 |
| São Jorge de Arroios | 15893,6 | 1,581 | nd | 0,27923 | 0,12207 | 0,87169 | 0,10996 | 4,29942 | 5 |
| São José | 8310,9 | 2,608 | nd | 0,36174 | 0,13110 | 0,86182 | 0,13536 | 7,50453 | 5 |
| São Miguel | 29495,4 | 2,184 | nd | 0,54944 | 0,09275 | 0,91243 | 0,13485 | 10,29879 | 5 |
| São Nicolau | 4674,5 | 1,648 | nd | 0,25794 | 0,20146 | 0,89782 | 0,08135 | 8,32075 | 5 |
| São Paulo | 6212,2 | 2,503 | nd | 0,40094 | 0,15982 | 0,86404 | 0,12588 | 8,98952 | 5 |
| São Sebastião da Pedreira | 5855,1 | 1,343 | nd | 0,17329 | 0,05535 | 0,81078 | 0,08422 | -2,27649 | 1 |
| São Vicente de Fora | 11176,3 | 1,588 | nd | 0,43687 | 0,08364 | 0,91301 | 0,13763 | 7,90566 | 5 |
| Sé | 7584,3 | 1,940 | nd | 0,34718 | 0,09780 | 0,82775 | 0,10684 | 3,31118 | 5 |
| Socorro | 27646,2 | 2,688 | 2,151 | 0,47900 | 0,27993 | 0,88076 | 0,17508 | 16,95291 | 5 |
| Apelação | 3962 | 1,582 | nd | 0,55692 | 0,15814 | 0,92951 | 0,23832 | 11,17551 | 5 |
| Camarate | 3496,5 | 1,146 | nd | 0,56748 | 0,11845 | 0,92540 | 0,17396 | 10,64837 | 5 |
| Prior Velho | 5406,6 | 1,249 | nd | 0,39078 | 0,12472 | 0,89384 | 0,13623 | 7,05814 | 5 |
| Sacavém | 4522,9 | 1,122 | nd | 0,41085 | 0,11847 | 0,90523 | 0,15091 | 6,34227 | 5 |
| Paço de Arcos | 4512 | 2,139 | nd | 0,25051 | 0,06294 | 0,88592 | 0,11009 | 1,09813 | 4 |
| Belas | 1142,2 | nd | 1,909 | 0,37863 | 0,05217 | 0,89123 | 0,12111 | 0,90155 | 4 |
| Queluz | 7229,3 | 1,466 | nd | 0,43988 | 0,11483 | 0,92628 | 0,16706 | 6,18645 | 5 |
| Alfragide | 7420,8 | 1,555 | nd | 0,20017 | 0,02181 | 0,87078 | 0,08221 | -3,61574 | 1 |
| Brandoa | 8014,7 | 1,599 | 2,009 | 0,57346 | 0,12581 | 0,92952 | 0,18830 | 11,50057 | 5 |
| Buraca | 9577,9 | 2,090 | nd | 0,48727 | 0,12369 | 0,91780 | 0,18423 | 7,14943 | 5 |
| Damaia | 14810,5 | 1,437 | nd | 0,43858 | 0,09950 | 0,90610 | 0,13897 | 4,84357 | 5 |
| Falagiera-Venda Nova | 9843,8 | 2,173 | 2,233 | 0,50256 | 0,07942 | 0,92001 | 0,16357 | 5,58795 | 5 |
| Mina | 6390,6 | 1,737 | nd | 0,45656 | 0,13156 | 0,91801 | 0,17141 | 6,93197 | 5 |
| Reboleira | 18902,2 | 1,413 | nd | 0,40571 | 0,13483 | 0,91496 | 0,16569 | 6,96633 | 5 |
| Odivelas | 11717,6 | nd | 1,436 | 0,37361 | 0,08457 | 0,91007 | 0,11423 | 3,56443 | 5 |
| Pontinha | 4970,7 | 1,141 | nd | 0,50499 | 0,10598 | 0,91131 | 0,15370 | 7,45231 | 5 |
| Almada | 12096,9 | 1,560 | nd | 0,41411 | 0,04667 | 0,89640 | 0,14907 | 3,27733 | 5 |
| Caparica | 1857,5 | 1,431 | nd | 0,47902 | 0,07788 | 0,91370 | 0,18370 | 5,81567 | 5 |
| Costa de Caparica | 1318,3 | 1,273 | nd | 0,36315 | 0,11894 | 0,87073 | 0,13908 | 5,84961 | 5 |
| Laranjeiro | 5415,2 | nd | 1,666 | 0,46020 | 0,08519 | 0,93044 | 0,18102 | 5,23984 | 5 |
| Trafaria | 993,4 | 1,143 | nd | 0,57673 | 0,08725 | 0,89468 | 0,20271 | 12,02343 | 5 |

**SOFTWARE ANALYSIS CODE**

###analysis for MDR

model1 <- casosMDR ~ f(area1, model='bym2', graph='graph')

res1<-inla(model1, family='poisson', E=eMDR, data=dt,

control.compute=list(dic=TRUE, waic=TRUE, cpo=TRUE), verbose=TRUE)

summary(res1)

install.packages("excursions")

library(excursions)

res1$.args$control.compute$config <- TRUE

result1 <- inla.rerun(res1)

ex_mdrTB <- excursions.inla(result1, u=0, type='!=', alpha=0.95)

table(ex_mdrTB$M)

table(contourmdrTB <- factor(

ex_mdrTB$M, c(0,-1,1), labels=c('sig<', 'nonsig', 'sig>')))

### analysis for non-MDR

model2 <- casosNONMDR ~ f(area1, model='bym2', graph='graph')

res2<-inla(model2, family='poisson', E=eNMDR, data=dt,

control.compute=list(dic=TRUE, waic=TRUE, cpo=TRUE), verbose=TRUE)

summary(res2)

library(excursions)

res2$.args$control.compute$config <- TRUE

result2 <- inla.rerun(res2)

ex_TB <- excursions.inla(result2, u=0, type='!=', alpha=0.95)

table(ex_TB$M)

table(contourTB <- factor(

ex_TB$M, c(0,-1,1), labels=c('sig<', 'nonsig', 'sig>')))

##associations with EDI

model3 <- casosMDR ~ f(area1, model='bym2', graph='graph')+EDIQ

res3<-inla(model3, family='poisson', E=eMDR, data=dt,

control.compute=list(dic=TRUE, waic=TRUE, cpo=TRUE), verbose=TRUE)

summary(res3)

round(res3$summary.fixed,3)

model4 <- casosNONMDR ~ f(area1, model='bym2', graph='graph')+EDIQ

res4<-inla(model4, family='poisson', E=eNMDR, data=dt,

control.compute=list(dic=TRUE, waic=TRUE, cpo=TRUE), verbose=TRUE)

summary(res4)

round(res4$summary.fixed,3)
